# Successful Amelioration of Mitochondrial Optic Neuropathy Using the Yeast *NDI1* Gene in a Rat Animal Model

**DOI:** 10.1371/journal.pone.0011472

**Published:** 2010-07-08

**Authors:** Mathieu Marella, Byoung Boo Seo, Biju B. Thomas, Akemi Matsuno-Yagi, Takao Yagi

**Affiliations:** 1 Department of Molecular and Experimental Medicine, The Scripps Research Institute, La Jolla, California, United States of America; 2 Doheny Eye Institute, Keck School of Medicine, University of Southern California, Los Angeles, California, United States of America; Case Western Reserve University, United States of America

## Abstract

**Background:**

Leber's hereditary optic neuropathy (LHON) is a maternally inherited disorder with point mutations in mitochondrial DNA which result in loss of vision in young adults. The majority of mutations reported to date are within the genes encoding the subunits of the mitochondrial NADH-quinone oxidoreductase, complex I. Establishment of animal models of LHON should help elucidate mechanism of the disease and could be utilized for possible development of therapeutic strategies.

**Methodology/Principal Findings:**

We established a rat model which involves injection of rotenone-loaded microspheres into the optic layer of the rat superior colliculus. The animals exhibited the most common features of LHON. Visual loss was observed within 2 weeks of rotenone administration with no apparent effect on retinal ganglion cells. Death of retinal ganglion cells occurred at a later stage. Using our rat model, we investigated the effect of the yeast alternative NADH dehydrogenase, Ndi1. We were able to achieve efficient expression of the Ndi1 protein in the mitochondria of all regions of retinal ganglion cells and axons by delivering the *NDI1* gene into the optical layer of the superior colliculus. Remarkably, even after the vision of the rats was severely impaired, treatment of the animals with the *NDI1* gene led to a complete restoration of the vision to the normal level. Control groups that received either empty vector or the GFP gene had no effects.

**Conclusions/Significance:**

The present study reports successful manifestation of LHON-like symptoms in rats and demonstrates the potential of the *NDI1* gene therapy on mitochondrial optic neuropathies. Our results indicate a window of opportunity for the gene therapy to be applied successfully after the onset of the disease symptoms.

## Introduction

Leber's hereditary optic neuropathy (LHON) is a hereditary optic atrophy that is characterized by an acute or subacute loss of central vision [1], [2]. By the time the disease is fully developed, the retinal ganglion cells die and the eyes and optic nerves become atrophied although a relative pupillary light reflex remains [3], [4]. Wallace *et al* reported for the first time that LHON was related to a point mutation in the mitochondrial DNA (mtDNA) [5]. Approximately 95% of LHON have one of three point mutations in the genes encoding subunits of the mitochondrial proton-translocating NADH-quinone oxidoreductase (complex I) which are G3460A (ND1), G11778A (ND4), and T14484C (ND6) [6]. Other complex I subunits known to be related to LHON include ND4L and ND5 [7].

In an effort to clarify mechanism of LHON and possible development of therapeutic strategies, animal models of LHON have been created in rodents. They were based on either direct exposure of complex I inhibitor into the eye [8], or transduction of iRNA for the NDUFA1 subunit of complex I in the mouse retina [9], and allotopic expression of the mutated human *ND4* (G11778A) gene in the rat retina [10]. These models exhibited degeneration of the retinal ganglion cell layer and the optic nerve of rodents, suggesting that complex I deficiencies in the retinal ganglion cells are associated with LHON symptoms. However, it is unclear whether LHON is somagenic or axogenic [11]. As described above, the available LHON animal models were raised by injection of causative factors (complex I inhibitor, iRNA for the NDUFA1 subunit or the mutated human *ND4* gene) into eyes. Here, we report a rat animal model that relied on the administration of complex I inhibitor, rotenone, in the optical layer of the superior colliculus (SC) of the brain where the retinal ganglion cells project their nerve terminals. The animals tested developed symptoms that resembled main clinical features of LHON documented so far.

At present, it seems difficult to repair mitochondrial DNA. Therefore, an allotopic strategy has been used to circumvent this problem. Since polypeptides encoded by mtDNA are highly hydrophobic, this may cause problems such as aggregation of the expressed polypeptides in cytoplasm and triggering immune response by over-expressed hydrophobic subunits in the cytoplasm. Regardless of defects of mtDNA- and nuclearDNA-encoded subunits of complex I, the alternative NADH dehydrogenase (Ndi1) from yeast mitochondria can restore NADH oxidase deficiencies and suppress reactive oxygen species (ROS) overproduction caused by complex I defects. In fact, we and other laboratories had previously demonstrated that the Ndi1 enzyme worked as a functional replacement for defective complex I in various mammalian culture cells [12]–[23]. In addition, the Ndi1 expression protected against complex I deficiencies of animals. So far, the *NDI1* gene has been shown to prevent or retard disease-like symptoms caused by complex I deficiencies [24]–[29]. In this paper, we report the first successful amelioration by the *NDI1* gene in a rat animal model.

## Results

### LHON-like features manifested in rats by administration of rotenone into the brain

**Figure 1:**
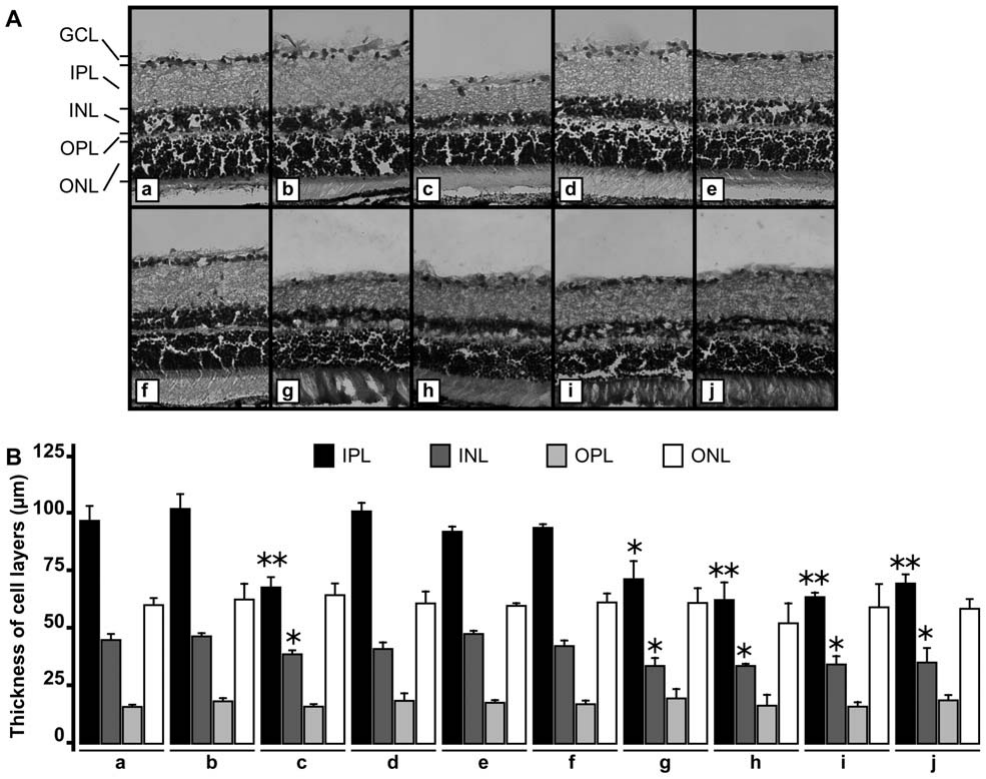
Representative images of the rat retina after rotenone exposure and the effect of rAAV5-NDI1. Microspheres loaded with rotenone was injected into the SC of rat brain. Two months later, the retina was examined for damage using hematoxylin-eosin staining. A, representative images of the retina; B, comparison of the cell layer thickness of the retina. The histogram shows the average of the thickness of the retina cell layers after hematoxylin-eosin staining (n = 4 for rats, n = 16 for retina slices from each rat retina). For both Figure 1A and 1B, a, control; b, rAAV5-NDI1 injection only; c, rotenone microspheres injection only; d, rAAV5-NDI1 injected immediately after the rotenone-beads administration; e, rAAV5-NDI1 injected 1 week after the rotenone microspheres administration; f, rAAV5-NDI1 injected 2 weeks postadministration of rotenone microspheres; g, rAAV5-GFP injected immediately after the rotenone microspheres administration; h, rAAV5-empty vector injected immediately after the rotenone microspheres administration; i, rAAV5-GFP injected 2 weeks after the rotenone microspheres exposure; j, rAAV5-empty vector injected 2 weeks after the rotenone microspheres administration. GCL, ganglion cell layer; IPL, inner plexiform layer; INL, inner nuclear layer; OPL, outer plexiform layer; ONL, outer nuclear layer. *p<0.05, **p<0.01, Student T-test compared to the same retina layer of control (a).

**Figure 2:**
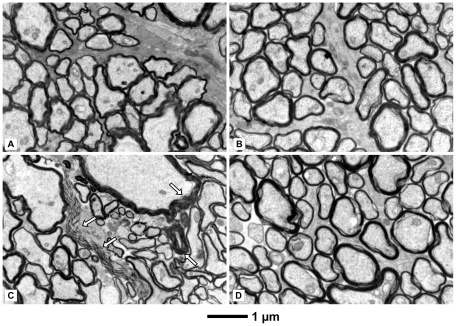
Electron micrographs of the optic nerves displaying the status of myelination. Rats were injected with rotenone-microspheres or rAAV5-NDI1 or both as described for Figure 1. The optic nerve was examined 1 month after the rotenone infusion. A: control (no injection). B: rAAV5-NDI1-injected. C: rotenone microspheres-injected. Parts of degenerated myelin are indicated by arrows. D: rotenone microspheres-treated with a simultaneous injection of rAAV5-NDI1.

We investigated the effect of rotenone administration into the rat brain and injection of an adeno-associated virus (type 5) carrying the *NDI1* gene (rAAV5-NDI1) by monitoring the histopathological hallmarks of the disease using Long Evans male rats. Rats were divided into 11 groups (Table 1). Both rotenone microspheres (3 µl per site) and viral particles (rAAV5-NDI1, rAAV5-GFP, or rAAV5-empty vector, 3 µl per site) were infused bilaterally in the optical layer of the SC of the rat brain. The eyes and brains were processed for immunohistochemistry 2 months after the initial treatments unless otherwise specified. The most common features of LHON, the optic nerve and retina atrophy, were identified in the rotenone-treated animals. Indeed, the microspheres releasing the complex I inhibitor elicited damage to the optic nerve and the retina. It should be noted that, unlike the subcutaneous injection of rotenone microspheres [25], no rotenone was detected in the blood stream of the animal. The retina of the rotenone-treated rats was significantly thinner than that of the control (Figure 1). The loss occurred mainly in the inner plexiform layer and the inner nuclear layer. The number of the retinal ganglion cells was also diminished compared to the control. The reduction of the retina thickness was not accompanied by any obvious inflammatory process. No immune cell infiltration was seen within the retina or the optic nerve. The optic nerve lost an average of 20% in diameter compared to the control (Figure S1). Electron microscopy of the optic nerve of rotenone-treated rats revealed disorganization of the neuronal fibers, axons of the retinal ganglion cells as well as some demyelination (Figure 2C).

**Table 1:** Animal groups and treatments.

Group	Treatment	Number of animals
None	rats did not receive any treatment	5
aav-GFP	rats were injected with rAAV5- GFP	4
aav-NDI1	rats were injected with rAAV5- NDI1	5
Rotenone	rats received an injection of rotenone microspheres	5
Rotenone + aav-NDI1	rats were injected with rAAV5-NDI1 2 days after the rotenone administration	5
Rotenone + aav-GFP	rats were injected with rAAV5-GFP 2 days after the rotenone administration	4
Rotenone + aav	rats were injected with rAAV5-empty vector 2 days after the rotenone administration	4
Rotenone-1w-aav-NDI1	rats received rAAV5-NDI1 injection 1 week after the rotenone administration	5
Rotenone-2w-aav-NDI1	rats received rAAV5-NDI1 injection 2 weeks after rotenone administration	5
Rotenone-2w-aav-GFP	rats received rAAV5-GFP injection 2 weeks after rotenone administration	4
Rotenone-2w-aav	rats received rAAV5-empty vector injection 2 week after rotenone administration	4

### Expression of Ndi1 in the retinal ganglion cells and the optic nerve in rats

**Figure 3:**
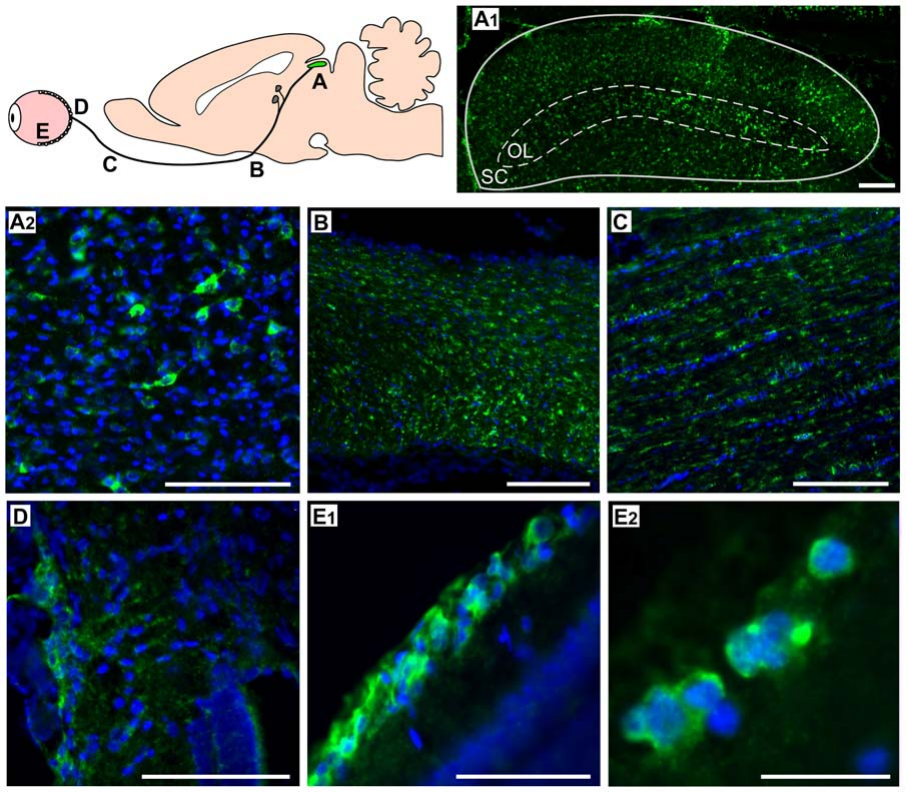
Expression of the Ndi1 protein throughout the optic nerve system. Rats received rAAV5-NDI1 in the SC. Two months after the injection, tissues were collected from the locations depicted in the cartoon (A – E) and immunohistochemically stained for the Ndi1 protein (green). Nuclei were visualized by DAPI (blue). A1: coronal section of the optical layer (OL) of the superior colliculus (SC). Scale bar  = 150 µm. A2: optical layer of the SC. Scale bar  = 40 µm. B: sagittal section of the optic chiasma. Scale bar  = 100 µm. C: sagittal section of the optic nerve. Scale bar  = 100 µm. D: entry point of the optic nerve into the retina. Scale bar  = 40 µm. E1: transversal section of the retina. Scale bar  = 40 µm. E2: a high magnification image of the retinal ganglion cell layer, Scale bar  = 10 µm.

Our objective was to achieve efficient introduction of the Ndi1 protein in the mitochondria of the whole retinal ganglion cell layer, axons, and the nerve terminals. We first attempted injections of rAAV5-NDI1 directly into the eye. However, this method was not reliable because the expression areas were dependent on the location of the injection and a complete spread throughout the entire retina was not always possible. On the contrary, injections of the same rAAV5-NDI1 to the SC where the terminals of the optic nerves are located gave rise to a consistent expression of Ndi1 in the entire region of our target. Within 2 months, Ndi1 was fully expressed in all regions of the SC, the optic nerve (including the chiasma and the entry point into the eye), and the retinal ganglion cell layer (Figure 3). It should be noted that we could detect Ndi1 as early as 1or 2 weeks after the rAAV5-NDI1 injection albeit at a much lower level (Figure S2). The Ndi1 expression matched the pattern of the mitochondrial network of the cell. The presence of Ndi1 did not result in any apparent change when compared to the control group in terms of the thickness of the retina (Figure 1B) and morphology of myelin sheath surrounding the axons (Figure 2B). The expression of Ndi1 was sustained for at least 6 months in rats and up to 20 months in mice.

### Protective action of Ndi1 against the deleterious effect of rotenone

**Figure 4:**
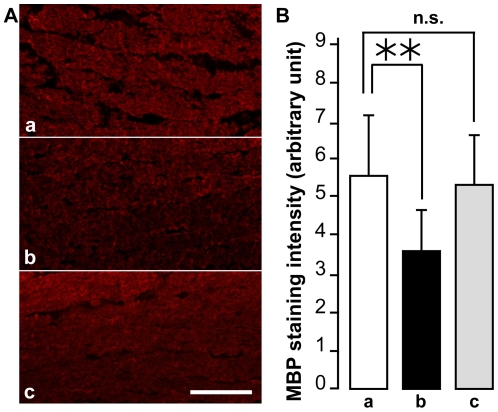
Loss of myelin in the optic nerve of rotenone-treated rats and its protection by rAAV5-NDI1. Sagittal sections of the optic nerve were obtained from the rats treated with rotenone for 8 weeks and were stained for myelin basic protein (MBP) using specific antibody. A: representative pictures of the MBP staining intensity. Scale bar  = 100 µm. B: histograms comparing the fluorescence intensity of MBP staining. MBP staining intensity was evaluated by ImageJ (NIH) with 8 measures per animal and 4 animals per group. **p<0.01, n.s. (no-significant) Student's T-test. Error bars represent the mean ± SD. For A and B: a, control; b, rats treated with rotenone for 8 weeks; c, rAAV5-NDI1 injected immediately after the rotenone microspheres.

First, we evaluated the effect of expressing Ndi1 on the morphological damage by rotenone. When the rats received rAAV5-NDI1 at the time of rotenone administration, the thickness of the retinal ganglion layer remained normal compared to control and the overall organization of the retina was conserved (Figure 1d). Similar protective effect was observed even when the rAAV5-NDI1 injection was done 1 or 2 weeks after the initiation of rotenone administration (Figure 1e and 1f). Similarly, no degeneration was identified in the structure of the optic nerve axon in the groups of rats injected with rAAV5-NDI1 in addition to rotenone (Figure 2d). In contrast, rAAV5-GFP or rAAV5-empty vector did not have any effect (Figure 1g, 1h, 1i, and 1j). Also, the rotenone exposure for 2 months resulted in a reduction of myelin basic protein (MBP) in the optic nerve by approximately 20% of control (Figure 4). The group of rats that received rAAV5-NDI1 retained the content of MBP.

### Restoration by Ndi1 of impaired vision of rotenone treated rats

**Figure 5:**
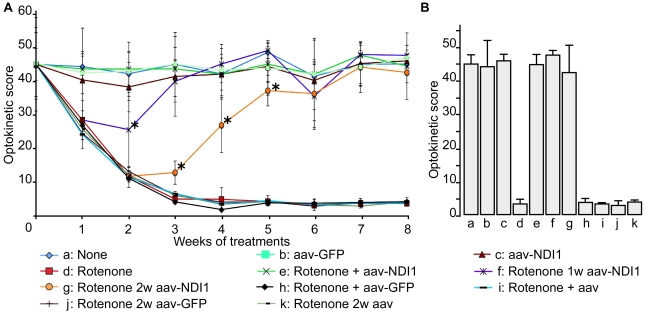
Optokinetic tests of rotenone-treated rats exhibiting loss of vision and the protective and ameliorative effects by Ndi1. Rats were injected with rotenone microspheres in the SC as described for Figure 1. Optokinetic tests were performed every week for the duration of the treatment. Each rat was monitored for 2 minutes and the time of head tracking response was counted to compute the optokinetic score. A: time dependent change of optokinetic scores of rats with various treatments. B: comparison of optokinetic scores of all rat groups at week 8. Treatments of the individual group of rats are as follows: a, control (no injection); b, control (rAAV5-GFP injection at week 0); c, rAAV5-NDI1 injection at week 0; d, rotenone injection at week 0; e, rotenone injection at week 0 and rAAV5-NDI1 at day 2; f, rotenone injection at week 0 and rAAV5-NDI1 injection at week 1; g, rotenone injection at week 0 and rAAV5-NDI1 injection at week 2; h, rotenone injection at week 0 and rAAV5-GFP injection at day 2; i, rotenone injection at week 0 and rAAV5-empty vector injection at day 2; j, rotenone injection at week 0 and rAAV5-GFP injection at week 2; k, rotenone injection at week 0 and rAAV5-empty vector injection at week 2. *p<0.05, ANOVA test excluding the rotenone-treated group.

Next, we examined the blindness of the rats by a T-maze behavior test (Figure S3). The non-treated rats (control group) had a strong preference to the dark side as expected. The group of rats that received rotenone still demonstrated a strong preference for the dark side. In other words, the damage caused by rotenone administration to the optic nerve and the retina was not sufficient to provoke total blindness in the rats. Therefore, a more sensitive test was used to evaluate their vision [30]. The testing involved monitoring for head tracking response with an animal placed in an optokinetic apparatus, and was performed at a fixed interval after rotenone administration (Movie S1). The rotenone group showed a gradual decrease of the head tracking score (time of head tracking in second) which reached the lowest level between the third and the fourth week of the treatment (Figure 5). The group injected with the rAAV5-NDI1 in addition to rotenone exhibited optokinetic scores that closely followed those of the control group. Remarkably, the animals that received the rAAV5-NDI1 injection 1 week after the rotenone administration increased their optokinetic scores and reached the level of the control group on week 4. A similar recovery of vision was achieved with the group that received the rAAV5-NDI1 injection 2 week after the rotenone treatment. In the latter case, a complete recovery took place on week 7. Injection of rAAV5-GFP or rAAV5-empty had no observable effect. Figure 5B summarized results of optokinetic experiments 8 weeks postinjection of rotenone. It is apparent that rAAV5-NDI1 can completely rescue the animals from vision loss but the control vectors (rAAV5-GFP or rAAV5-empty vector) cannot. This is the first report in which the *NDI1* gene was shown to cure symptoms caused by complex I deficiencies in an animal model. These results strongly suggest the great potential of the *NDI1* gene as a therapeutic agent for vision loss caused by complex I deficiency. It was suspected that, at earlier stages (1–2 weeks) of the rotenone treatment, there is already some damage to the retina or the optic nerve that causes impairment of vision but that damage is still restorable. We have examined tissues of rats 2 and 4 weeks after rotenone microspheres injection. No cell death was detected in the ganglion cell layer of the retina of rats 2 weeks after rotenone injection, but significant cell death was detected in rats 4 weeks post injection of rotenone (Figure S4). In addition, we looked for a sign of ROS formation by visualizing oxidized DNA. Both the retina and the optic nerve showed positive results after 2 weeks of rotenone exposure (Figure S5). This agrees with our previous observations that, both *in vitro* and *in vivo*, generation of ROS was one of the earliest events caused by complex I inhibition [25], [31].

### Prevention by Ndi1 of the cell death within the ganglion cell layer and the SC

**Figure 6:**
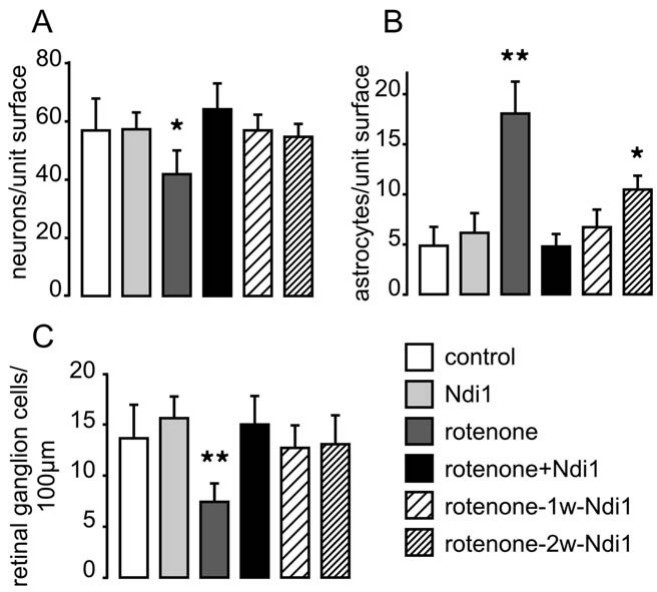
Loss of neurons and retinal ganglion cells caused by rotenone treatment. Rats were subjected to rotenone exposure for 2 months as detailed in the legend to Figure 1. A and B: Tissue sections of the optical layer of the SC were stained with specific antibodies targeting neurons (NeuN) and astrocytes (GFAP), respectively. C: after hematoxylin-eosin staining, the retinal ganglion cells of the GCL were counted. Cells were counted per field of view in 64 different sections separated by at least 100 µm. *p<0.05, **p<0.001, one way ANOVA test. Error bars represent the mean±SD.

The brain slices of the rats, sacrificed 2 months after rotenone administration, were stained with neuron specific antibody NeuN and the number of neuron cells remaining in the optic layer of the SC was counted (Figure 6A). In all animals tested, only the rotenone group showed a significantly reduced number of neurons. In parallel, we estimated the amount of astrocytes using antibody against the glial fibrillary acidic protein (GFAP) (Figure 6B). The rotenone group had a higher number of astrocytes in the SC compared to others. A significant increase of GFAP positive cells in samples of the rotenone-2w-aav-NDI1 animals group is also noticed. The number of retinal ganglion cells was assessed for all animals used in this study. After hematoxylin-eosin staining of the sagittal section of the eyes, retinal ganglion cells were counted within an area spanning 100 µm. Those areas were randomly chosen covering the entire retina. Figure 6C shows that the Ndi1 expressing animals did not lose cells whereas the group of animals treated with only the complex I inhibitor lost approximately 55% of the retinal ganglion cells when compared to the control group.

**Figure 7:**
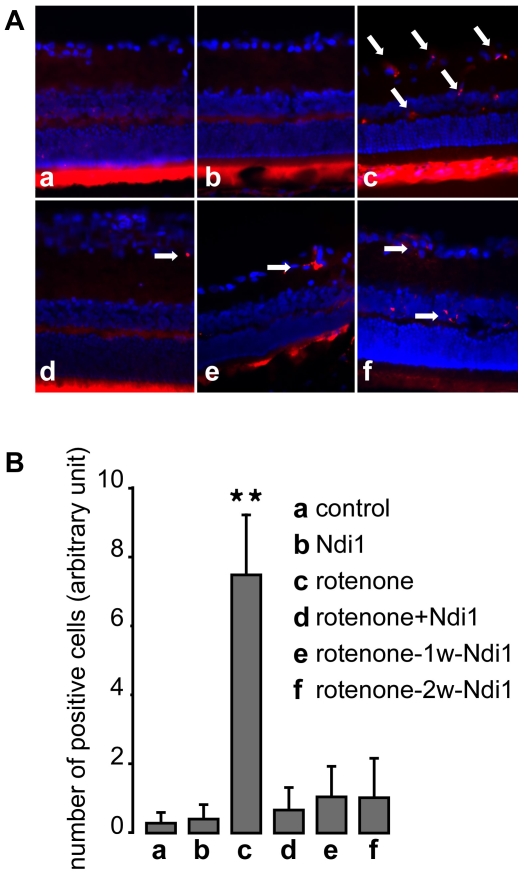
Prevention of apoptosis of cells treated with rotenone by Ndi1 expression. Rats were subjected to rotenone exposure for 2 months as detailed in the legend to Figure 1 and the retina samples were stained for single strand DNA (red) and DAPI (blue). A: (a) control (no injection), (b) rAAV5-NDI1 injected animal, (c) rotenone microspheres injected animal, (d) rAAV5-NDI1 was injected immediately after the rotenone administration, (e) rAAV5-NDI1 was injected 1 week after the rotenone administration, (f) rAAV5-NDI1 was injected 2 weeks after the rotenone administration. The arrows highlight some of the cells carrying the apoptotic marker. Bright fluorescence seen in the cones and rods layer of the retina is un-specific and is not considered for ssDNA evaluation. B: The number of retinal ganglion cells positive for ssDNA staining was compiled in a histogram. Cells were counted per field of view in different sections separated by at least 100 µm. **p<0.0001, ANOVA test. Error bars represent the mean±SD.

It has been shown that complex I dysfunction is related to the cell apoptosis driven by mitochondria. In order to determine the type of cell death occurring in the retina after rotenone treatment, we searched for apoptosis hallmarks in the tissue samples. The retina was stained with antibodies specific for the cleaved active form of caspase 3, the cleaved form of caspase 9 or single-stranded DNA (ssDNA). Figure 7 clearly shows a higher number of the apoptotic marker (ssDNA) within the retina of the rotenone-treated group whereas no or few positive staining was seen in the other animals (see also Figure S6).

### Retention of ATP and suppression of ROS generation by Ndi1 in RGC-5 cell line

**Figure 8:**
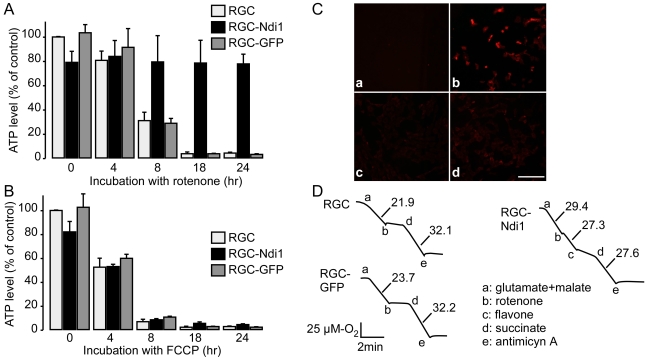
Compensatory effect by Ndi1 on complex I inhibition in RGC-5 cells. A and B, cellular ATP level. RGC-5 cells with or without expressed Ndi1 or GFP were grown in 6 well plates and incubated for the indicated time with 100 nM rotenone (A) or 1 µM FCCP (B). The ATP content of the cells was evaluated using the luciferase assay system. Error bars represent the mean±SD. C, MitoSox staining of RGC-5 cells incubated with 100 nM of rotenone for 8 hours. RGC-5 cells were incubated in the absence (a) or presence (b) of the complex I inhibitor. RGC-5 cells expressing Ndi1 were incubated in the absence (c) or presence (d) of rotenone. D, Oxygraphs showing the respiratory activity of RGC-5 cells. Cells were permeabilized by digitonin treatment. The measurements were performed with 1×10^7^ cells per assay. Respiratory substrates and inhibitors were added sequentially at the concentrations given in Materials and Methods. The rate of oxygen consumption is shown in µM O_2_/min.

We next explored the physiological basis for the observed protective effects exerted by Ndi1 expression using an *in vitro* system. A retinal ganglion cell line (RGC-5 cells) stably expressing Ndi1 was established for this purpose. When the RGC-5 cells were incubated in the presence of rotenone, the cellular ATP content dropped significantly within 8 hours and reached almost zero in 18 hours. The Ndi1-expressing RGC-5 cells exhibited a somewhat reduced level of ATP but were able to sustain that level in the presence of rotenone (Figure 8A). On the contrary, and in a predicted manner, FCCP, an uncoupler that dissipates proton gradient, rapidly exhausted ATP regardless of the presence of Ndi1 (Figure 8B). Furthermore, RGC-5 cells incubated with rotenone gave rise to ROS production as illustrated in Figure 8C. The cells expressing Ndi1, however, showed no detectable ROS production. Finally, the respiratory activity was assessed by measuring mitochondrial consumption of oxygen using digitonin-permeabilized cells (Figure 8D). Addition of complex I-dependent substrates, glutamate plus malate, to the RGC-5 cells expressing Ndi1 accelerated the respiration rate and the enhanced activity was not inhibited by rotenone. The addition of succinate, a substrate for complex II, generated about the same respiration rate between the control and Ndi1-expressing cells. In all measurements, the RGC-5 cells expressing the GFP protein behaved exactly the same as the non-transduced control cells.

## Discussion

The animal models of LHON reported so far dealt with the retinal ganglion cell body as the target [8], [10]. It is currently unknown whether the disease is somagenic or axogenic [11]. Use of microspheres allows a slow, persistent and long-term release of rotenone. Our earlier study indicated that a subcutaneous injection of rotenone-loaded microspheres into rats resulted in a sustained level of rotenone in the blood for at least 2 months [25]. When the rotenone-loaded microspheres were injected into the SC, the rotenone effect was observed in the entire population of the ganglion cells in the retina. There are at least two points that need to be discussed in relation to the actual disease. One is that the rats treated with rotenone did not show complete blindness because they were still able to distinguish between the light and the dark environment in the T-maze test. This is consistent with the observation that, in human LHON, patients suffering from degraded visual acuity are able to preserve a pupillary reflex to the light [3], [4]. The other point is that, after 2 weeks of exposure to rotenone, the rats severely lost their vision with no discernible damage to the ganglion cell layer. At this stage, there was an indication of ROS generation in both the retina and the optic nerve axon. This is presumably caused by the action of rotenone but did not seem fatal to the cells. Delivering the *NDI1* gene at this point restored the vision to the normal level within several weeks. These results are especially important in light of the fact that spontaneous recovery of visual acuity does occur in some LHON patients (but rare) even years after the onset of the disease [6], [32]–[34]. There are two distinct (acute and atrophic) phases in LHON. In the acute phase, the blurring of vision appears, the perception of colors is altered, and visual acuity of the patients is severely reduced. After the acute phase, the retinal nerve fiber layer degenerates, the eyes and optic nerve becomes atrophied, and vision remains severely impaired in the atrophic phase [35], [36]. Carelli *et al*. [6] reported oligodendrocytic cytoplasmic tongues wrapping denuded axons, strongly suggestive of ongoing axonal remyelination in the patients with restored vision. These findings illustrate a dynamic process occurring with the surviving axons in LHON and may well be represented in our rat model. These results indicate that there exists a window of opportunity for the gene therapy to be applied successfully before the loss of cells.

It is well recognized that defects of complex I lead to two consequences: (1) decrease of the membrane potential across the inner mitochondrial membrane and the resultant impairment of ATP synthesis; (2) ROS overproduction that causes oxidative stress and apoptosis [21], [37]–[39]. At the present time, it is unclear which is the major event in the retinal ganglion cells in LHON. Our *in vitro* experiment using the RGC-5 line showed a severe loss of ATP, which agrees with the results reported for cellular models of LHON [40], [41]. On the other hand, a number of studies, including our own, suggested involvement of apoptosis in which ROS overproduction plays a critical role [21], [42]. As we demonstrated previously, the Ndi1 enzyme is able not only to restore electron transfer pathway but to suppress ROS overproduction from defective complex I [21], [31]. In other words, the dual role of Ndi1 makes it an effective therapeutic molecule regardless of the nature of the link between complex I dysfunction and manifestation of the disease. In addition, it is conceivable that the ATP level sustained by Ndi1-linked respiration is enough to prevent dysfunction caused by complex I defects in retinal ganglion cells.

One of the important findings in this paper is that the *NDI1* expression restored vision to the animals in our rat model. This fact might stimulate a possibility of transkingdom gene therapy which has never been explored. Our preliminary results (Marella, M. et al., unpublished results) suggest that the Ndi1 expression scarcely stimulate inflammatory or immune response in rats or nonhuman primates (see reviews [12], [43], [44]). It is generally accepted that transgene expression mediated by an adeno-associated virus takes several weeks before reaching an optimal level [45]. In this study, it was noticed that there was a sign of recovery of the vision as early as 1 to 2 weeks after the rAAV5-NDI1 injection into the animals that were displaying severe impairment by rotenone treatment. The level of Ndi1 expression 1 week after the rAAV5-NDI1 injection was considerably lower than that observed 1 or 2 months after the injection. This may imply that a small amount of Ndi1 might be sufficient to compensate for the reduced complex I activity. In addition, the Ndi1 protein seems to have a long lasting expression in the retinal ganglion cells as we were able to detect it even 6 months after the injection. Together with the fact that the adeno-associated virus strategy was successfully used to treat retinal degeneration in human [46]–[48], it is conceivable that use of the *NDI1* gene may prove to be a successful gene therapy to treat visual loss linked to mitochondrial optic neuropathy.

## Materials and Methods

### Animals and treatments

Eight weeks old Long Evans male rats (Charles River, Reno) were housed 2 per cage in a 12 hours light/dark cycle and were fed with food and water *ad libitum*. All procedures were approved by the Institutional Animal Care and Use Committee (IACUC) at The Scripps Research Institute (approval ID 08-0065). Stereotaxic injections were performed under isoflurane anesthesia. Three µl (per site) of rotenone microspheres or 3 µl (per site) of rAAV5-NDI1 (serotype 5, 3.1×10^12^ genome copy/ml, Applied Viromics) were infused at 350 nl/min into the optical layer of the SC bilaterally (coordinates for the Bregma: antero-posterior −0.63 cm; media-lateral ±0.15 cm; dorso-ventral −0.36 cm). rAAV5-NDI1 was prepared as described previously [16]. As control vectors, rAAV5-GFP and rAAV5-empty (serotype 5, 3.1×10^12^ genome copy/ml, Applied Viromics) were also prepared following the preparation method of rAAV5-NDI1.

### Rotenone microspheres production

Biodegradable microspheres containing rotenone were prepared as described previously [25] with modifications. Briefly, 200 mg of rotenone was dissolved with 200 mg of poly(DL-lactide-co-glycolide) (Sigma, lactide:glycolide 75∶25, mol wt 90,000–126,000) in 7 ml of dichloromethane. The organic phase was poured into ice-cold 8% (w/v) polyvinyl alcohol (hot water soluble; Sigma). The microspheres were collected by decantation and only the top layer of the microspheres was retrieved and washed with sterile distilled water. The average size of the beads was estimated to be ∼8 µm.

#### Behavioral studies

The T-maze test was used to evaluate the capacity of the rats to distinguish between dark and light fields (www.ratbehavior.org/RatsAndMazes.htm). The maze was placed under bright light and one of the ends was covered to create a dark spot in such way that the animal is allowed to choose between the light and the dark side. Each animal was tested 15 times (3 each at 5 separate times) with occasional swapping of the two sides to prevent preferences. The optokinetic test was performed to assess the vision of the animals in a scalable manner [30]. This test consisted of placing a rat in a squared transparent cage surrounded by 4 monitors linked to a computer. The monitors displayed a pattern of vertical black and white strips which move from side to side at a defined speed. The screens were 800 pixels wide. The black and white strips were 60 and 75 pixels wide, respectively. The direction was switched every 4 seconds. The head tracking response of the rat was monitored for 2 minutes. Each test was videotaped for analysis (see Movie S1 for an example).

### Electron microscopy

Rats were euthanized, under a high dose of Pentothal, by transcardiac perfusion of phosphate buffered saline (PBS) followed by a fixative consisting of 1% paraformaldehyde with 3% glutaraldehyde in 0.1 M cacodylate with the addition of 1 mM calcium chloride. The eyes were removed and continued with immersion fixation in the same fixative on ice. The optic nerve and eyecup were dissected free of muscles, washed in a buffer, and postfixed in 1% OsO_4_ in 0.1 M cacodylate buffer with 3.5% sucrose. Tissues were subsequently washed in distilled water, dehydrated in a graded ethanol series followed by transitioning in propylene oxide and embedded in Embed 812/Araldite (Electron Microscopy Sciences, Hatfield PA). Initially, thick (1.5 µm) sections of the optic nerves were cut and stained with toluidine blue for light microscopy assessment. Subsequently, transverse thin sections (60 nm) of the optic nerves were cut with a diamond knife (Diatome, Hatfield PA), mounted on copper slot grids coated with parlodion and stained with uranyl acetate and lead citrate for examination on a Philips CM100 electron microscope (FEI, Hillsbrough OR). Images were documented using a Megaview III ccd camera (Olympus Soft Imaging Solutions, Lakewood CO).

### Histological analysis

Rats were euthanized under a high dose of Pentothal, by transcardiac perfusion of PBS following by 4% paraformaldehyde (v/w in PBS, pH 7.4). The brains and eyes were retrieved and cryoprotected, overnight at 4°C, in 20% sucrose (v/w in PBS, pH 7.4). Tissue samples were sliced using a cryostat at 30 µm (brains) or 12 µm (eyes). Each slice was collected onto Superfrost slide (Fisher) and stored at −20°C. For immunohistological procedures, antigen retrieval was carried out as follows: the slides were placed in a preheated solution of 1 mM EDTA, 10 mM Tris-Cl, pH 8 and micro-waved for 15 minutes at 85–90°C. The solution was cooled down to room temperature in 10 minutes and the slides were transferred to PBS for staining. The samples were blocked using Tris buffered saline (TBS, pH 7.8) containing 10% horse serum, 0.1% Triton X-100 for 1 hour and then incubated overnight at 4°C with primary antibodies in TBS, 10% horse serum, 0.1% Triton X-100. Antibodies used are Ndi1 (1/500), NeuN (1/200); GFAP (1/400); ssDNA (1/200), and cleaved caspase3/9 (1/400) from Cell Signaling; MBP (1/200) from Chemicon; 7,8-dihydro-8-oxo-deoxyguanine (8-oxo-dG) (1/300) from Cosmo Bio Co, Japan. After 3 washes with PBS, secondary antibodies were applied on the section for 2 hours at room temperature. The Ndi1 antibody was revealed with a specific horseradish peroxidase conjugated antibody (1/1000; Calbiochem) and the TSA Plus fluorescein system kit (PerkinElmer). Histochemical staining for 8-oxo-dG (1/300 dilution, Cosmo Bio Co., Japan) was performed as described before [25] with minor modifications: endogenous peroxidase activities were eliminated by incubation of the tissue samples in methanol 3% H_2_O_2_ for 30 min and the Tyramide Signal Amplification kit (TSA, Amersham) was used according to the manufacturer's instructions.

### Hematoxylin-Eosin staining

H&E staining was performed on frozen section as described previously [20]. The sections (10–30 µm) on slides were immersed in xylene (10 min, twice), and rehydrated in a decreasing ethanol series diluted in distilled water (100%, 100%, 95%, 95%, 75%, 0%, 1 min each). The sections were rinsed in deionized water, stained in hematoxylin for 45 sec, rinsed in deionized water, and finally stained in eosin for 1 sec. After the color reaction, sections were dehydrated through an ethanol series into xylene and mounted using Permount mounting medium (Fisher Scientific, Pittsburgh, PA).

### NADH dehydrogenase activity staining

Eye slices were incubated in a solution containing 2 mg/ml nitro blue tetrazolium, 0.5 mM NADH, 50 mM Tris-Cl, pH 7.6 at 37°C for 15 min. The tissues were washed 2 times with TBS and destained with a series of increasing concentration of acetone solutions (30%, 60%, 90%). The slices were dried and mounted with Permount (Fisher).

### ATP measurements and MitoSox staining on RGC-5 cells

The RGC-5 cell line, kindly provided by Dr. Agarwal of UNT Health Science Center [49] were maintained in DMEM 10% horse serum at 37°C, 5% CO_2_. For the experiments, the cells were cultured in 6 well plates and incubated under the conditions described in the result section. The cells were scraped in PBS poured into centrifuge tubes and the total protein concentration was determined. For ATP measurements, ice cold perchloric acid (2M, 500 µl) was added to the cell suspension and, after 5 minutes, 175 µl of 1M Bicine and 306 µl of potassium hydroxide (4M) were added. The tubes were kept on ice for 15 minutes. After centrifugation, the ATP concentration was determined on the supernatant using the ATP Determination Kit (Invitrogen). Production of reactive oxygen species (ROS) in mitochondria was visualized by MitoSox (Invitrogen) staining using live cells as follows: cultured RGC-5 cells were incubated with or without 100 nM of rotenone for the indicated period of time. The MitoSox reagent was diluted to a final concentration of 1.5 µM in warm DMEM 1% serum and added to the cells. After 10 minutes, cells were fixed with 4% paraformaldehyde for 10 minutes, mounted onto glass slides with Mowiol mounting medium, and observed under a Zeiss fluorescence microscope by using excitation 510 nm/emission 580 nm.

### Oxygen consumption measurements on RGC-5 cells

The cells were harvested by trypsinization and resuspended in 1 ml of a medium containing 20 mM Hepes, pH 7.1/250 mM sucrose/10 mM MgCl_2_. The cells were treated with digitonin until more than 90% of the cells are stained by trypan blue. The digitonin-treated cells were washed with the same medium. Oxygen consumption was measured polarographically in 0.6 ml of a buffer containing 20 mM Hepes, pH 7.1/250 mM sucrose/10 mM MgCl_2_ by using a Clark electrode in a water-jacketed chamber maintained at 37°C. Concentrations of substrates and inhibitors used were: 5 mM glutamate, 5 mM malate, 5 µM rotenone, 5 mM succinate, 5 µM antimycin A, and 0.5 mM flavone.

### Analyses

The image intensity was analyzed by ImageJ (NIH). Statistic analyses of the experimental data were performed by using appropriate test from Student t test, one-way or post hoc ANOVA.

## Supporting Information

Figure S1 Effect of rotenone administration on the diameter of the optic nerve. Rats were subjected to rotenone exposure for 2 months as detailed in the legend to Figure 1. A: Representative pictures of sections of the optic nerve stained with hematoxylin-eosin. B: The diameter of the largest portion of each tissue was determined and compiled into histograms. *p<0.05, Student's T-test. Error bars represent the mean±SD.
(2.67 MB TIF)

Figure S2 Expression of the Ndi1 protein in the retinal ganglion cells and the optic nerve of the rat 1 week and 1 month after receiving rAAV5-NDI1. The presence of Ndi1 was evaluated by the NADH dehydrogenase activity and TSA-enhanced antibody staining of the retina and the optic nerves of rats. Tissue samples were collected 0, 1 week, and 1 month after injection of rAAV5-NDI1 into the superior colliculus. Scale bar  = 50 µm. Histograms on the right compare the number of RGC that are expressing Ndi1 between the1 week and the 1 month retina samples. *p<0.05, Student's T-test. Error bars represent the mean±SD.
(2.10 MB TIF)

Figure S3 Behavioral study to assess vision of treated rats. Rats were subjected to rotenone exposure for 1 month as detailed in the legend to Figure 1. The animals were evaluated for their capacity to distinguish the dark and the light environment. In a bright room, animals were placed in a T-maze in which one of the ends was covered to create a dark section. Normal rats rush to the dark section in order to escape from the stress of the bright environment. Error bars represent the mean±SD.
(0.07 MB TIF)

Figure S4 Time-dependent induction of apoptosis in the ganglion cell layer by rotenone administration. Rats were subjected to rotenone exposure as detailed in the legend to Figure 1. The retina was stained for TUNEL (green) or DAPI (blue). Arrows highlight the TUNEL-positives nuclei. Scale bar  = 40 µm.
(1.12 MB TIF)

Figure S5 Early events of ROS generation caused by rotenone exposure. Rotenone-loaded microspheres were infused bilaterally in the optical layer of the superior colliculus of the rat brain. Two weeks after the injection, the optic nerve and the eyes were processed for histochemical assays. DNA oxidation in the respective tissues was assess by histochemical staining using antibody against 8-oxo-dG (green). Nuclei were visualized with DAPI (blue). Oxidative damage can be seen in the tissues from rotenone-treated rats. Scale bar  = 15 µm.
(0.53 MB TIF)

Figure S6 Caspase activation in ganglion cell layer induced by rotenone treatment. Rats were subjected to rotenone exposure for 2 months as detailed in the legend to Figure 1. The retina of the rats that received rotenone microspheres injection in the SC was stained for ssDNA and cleaved forms of caspase. (a) ssDNA, (b) cleaved form of caspase, (c) DAPI, (d) merge of the three images. Control rats that received no rotenone did not exhibit positive signals (not shown). Scale bar  = 40 µm.
(0.51 MB TIF)

Movie S1 Optokinetic tests comparing between a rat treated with roteone and a rat that received Ndi1 in addition to rotenone treatment.
(3.80 MB MOV)
