# Supplementary figures and images for: Successful Amelioration of Mitochondrial Optic Neuropathy Using the Yeast NDI1 Gene in a Rat Animal Model
Source: PLoS One. 2010 Jul 8;5(7):e11472. doi: 10.1371/journal.pone.0011472 (PMC2900204; doi:10.1371/journal.pone.0011472)

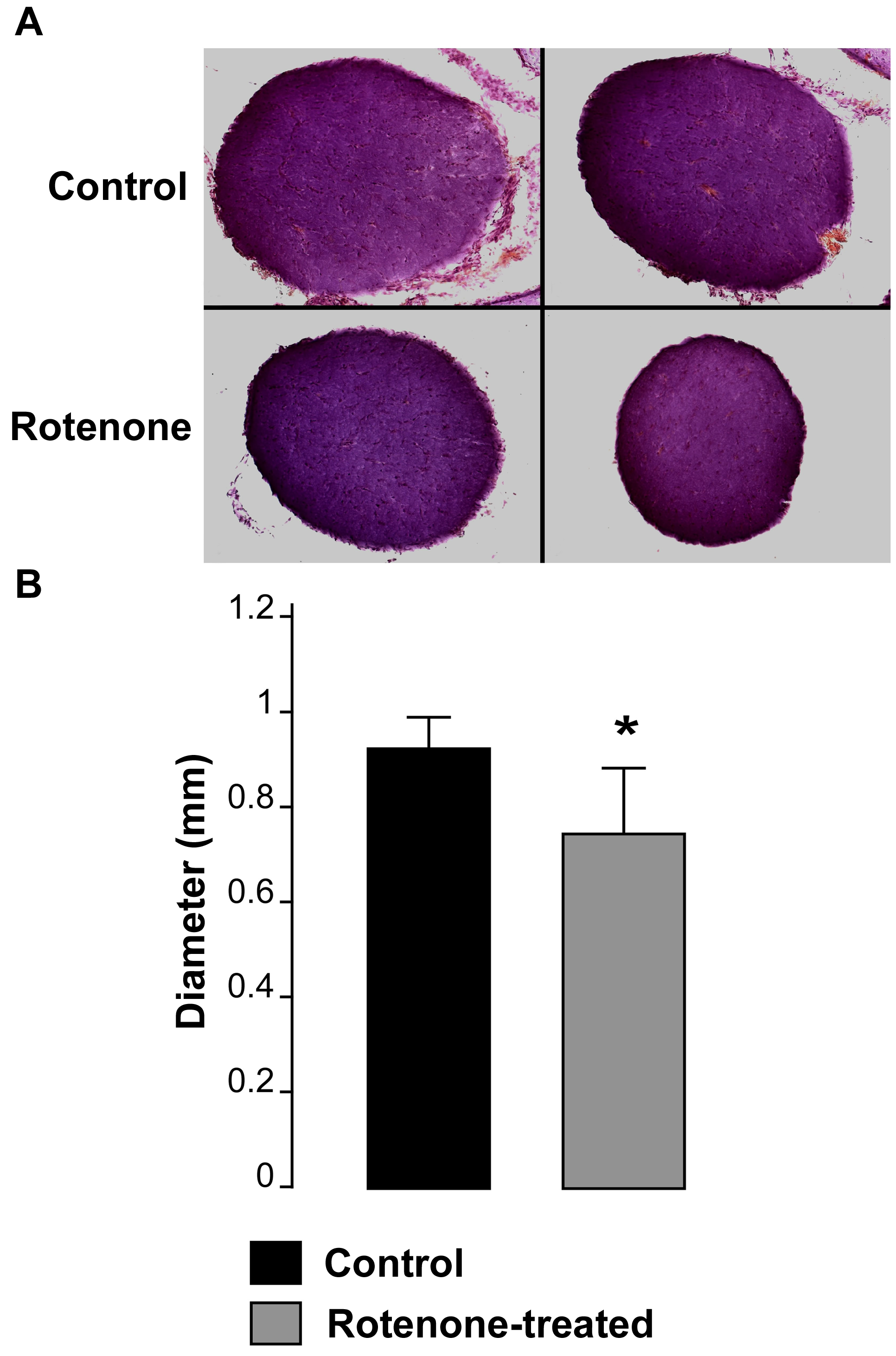

Supplement: Figure S1 — Effect of rotenone administration on the diameter of the optic nerve. Rats were subjected to rotenone exposure for 2 months as detailed in the legend to Figure 1. A: Representative pictures of sections of the optic nerve stained with hematoxylin-eosin. B: The diameter of the largest portion of each tissue was determined and compiled into histograms. *p<0.05, Student's T-test. Error bars represent the mean±SD. (2.67 MB TIF) [file pone.0011472.s001.tif]

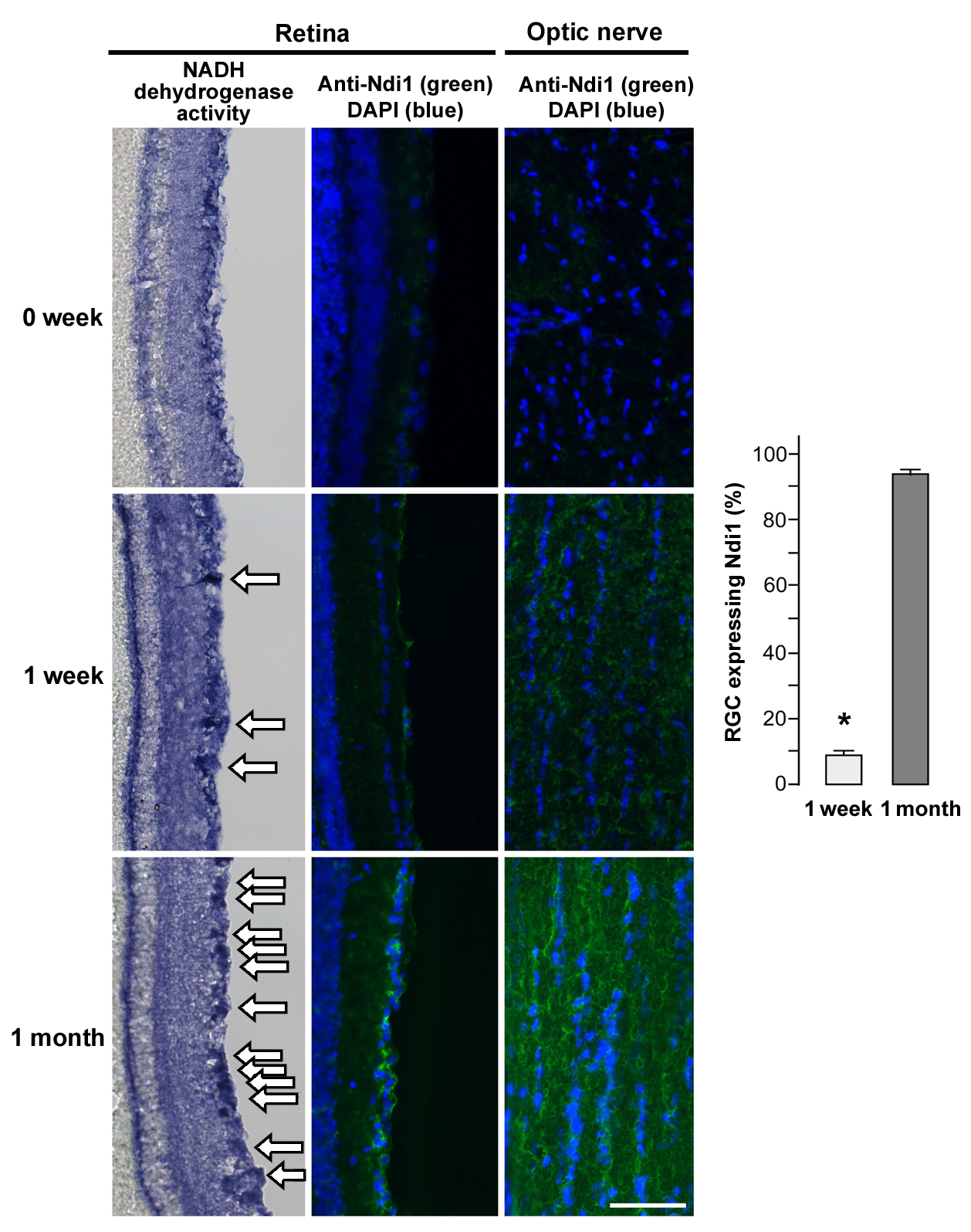

Supplement: Figure S2 — Expression of the Ndi1 protein in the retinal ganglion cells and the optic nerve of the rat 1 week and 1 month after receiving rAAV5-NDI1. The presence of Ndi1 was evaluated by the NADH dehydrogenase activity and TSA-enhanced antibody staining of the retina and the optic nerves of rats. Tissue samples were collected 0, 1 week, and 1 month after injection of rAAV5-NDI1 into the superior colliculus. Scale bar = 50 µm. Histograms on the right compare the number of RGC that are expressing Ndi1 between the1 week and the 1 month retina samples. *p<0.05, Student's T-test. Error bars represent the mean±SD. (2.10 MB TIF) [file pone.0011472.s002.tif]

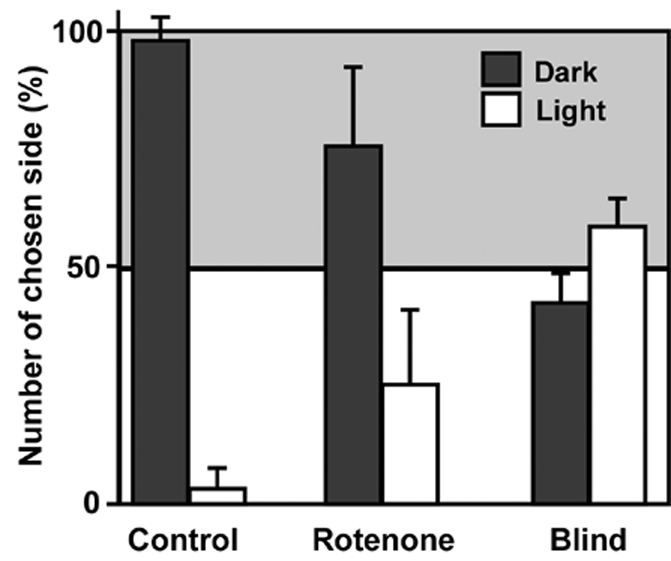

Supplement: Figure S3 — Behavioral study to assess vision of treated rats. Rats were subjected to rotenone exposure for 1 month as detailed in the legend to Figure 1. The animals were evaluated for their capacity to distinguish the dark and the light environment. In a bright room, animals were placed in a T-maze in which one of the ends was covered to create a dark section. Normal rats rush to the dark section in order to escape from the stress of the bright environment. Error bars represent the mean±SD. (0.07 MB TIF) [file pone.0011472.s003.tif]

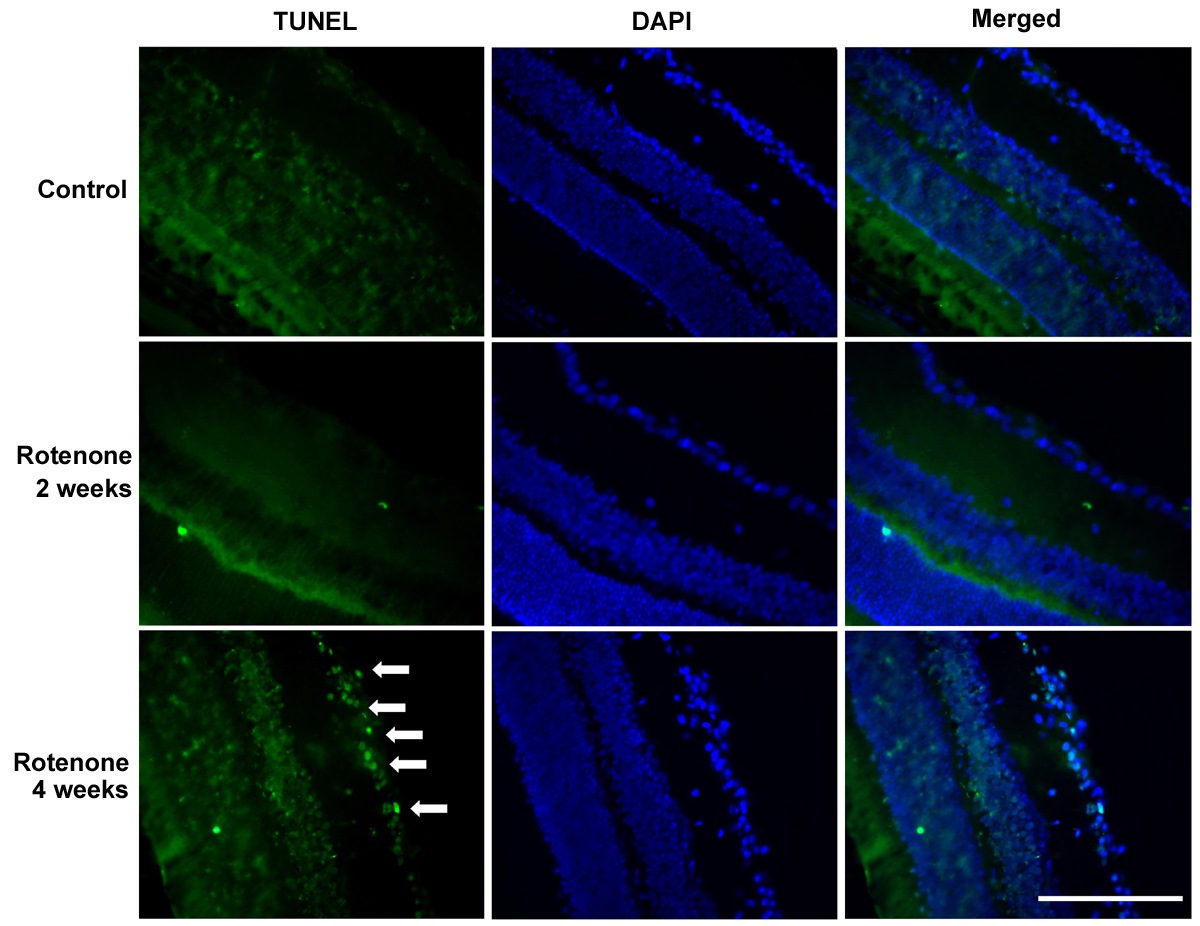

Supplement: Figure S4 — Time-dependent induction of apoptosis in the ganglion cell layer by rotenone administration. Rats were subjected to rotenone exposure as detailed in the legend to Figure 1. The retina was stained for TUNEL (green) or DAPI (blue). Arrows highlight the TUNEL-positives nuclei. Scale bar = 40 µm. (1.12 MB TIF) [file pone.0011472.s004.tif]

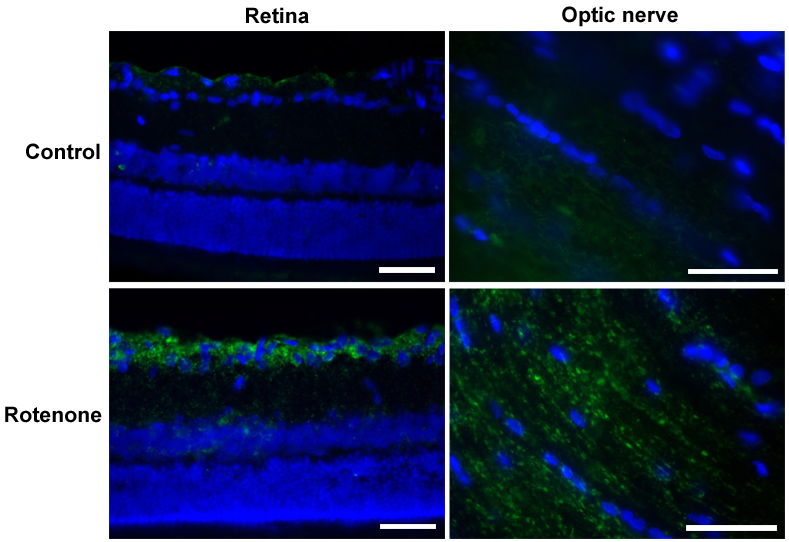

Supplement: Figure S5 — Early events of ROS generation caused by rotenone exposure. Rotenone-loaded microspheres were infused bilaterally in the optical layer of the superior colliculus of the rat brain. Two weeks after the injection, the optic nerve and the eyes were processed for histochemical assays. DNA oxidation in the respective tissues was assess by histochemical staining using antibody against 8-oxo-dG (green). Nuclei were visualized with DAPI (blue). Oxidative damage can be seen in the tissues from rotenone-treated rats. Scale bar = 15 µm. (0.53 MB TIF) [file pone.0011472.s005.tif]

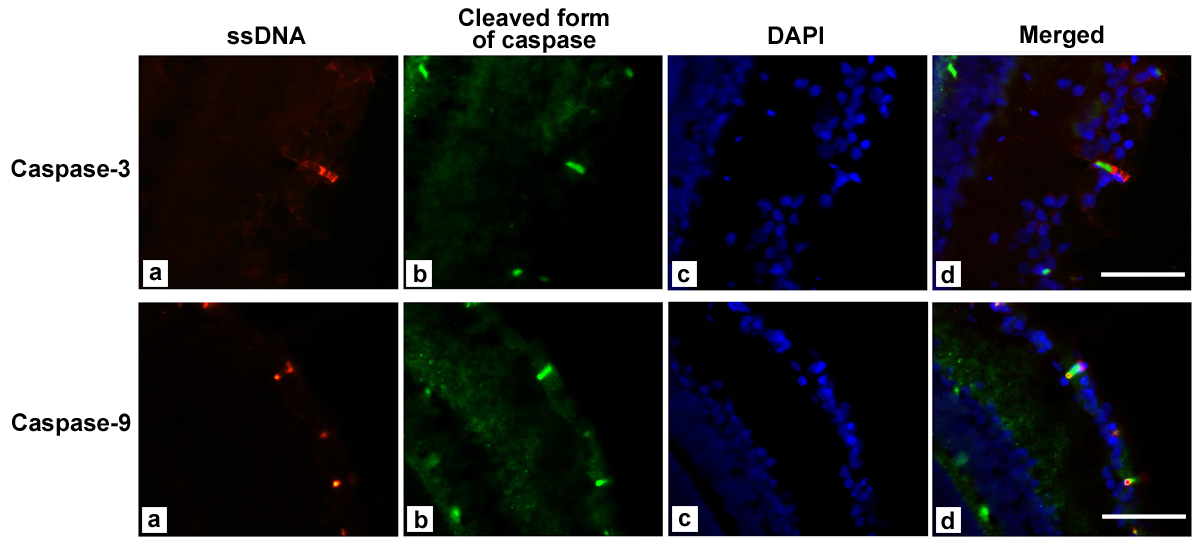

Supplement: Figure S6 — Caspase activation in ganglion cell layer induced by rotenone treatment. Rats were subjected to rotenone exposure for 2 months as detailed in the legend to Figure 1. The retina of the rats that received rotenone microspheres injection in the SC was stained for ssDNA and cleaved forms of caspase. (a) ssDNA, (b) cleaved form of caspase, (c) DAPI, (d) merge of the three images. Control rats that received no rotenone did not exhibit positive signals (not shown). Scale bar = 40 µm. (0.51 MB TIF) [file pone.0011472.s006.tif]
